# Multi‐omics integration highlights transposable elements as latent regulatory factors involved in the preclinical processes of Alzheimer's disease and related dementias‐ADRD

**DOI:** 10.1002/alz70855_106069

**Published:** 2025-12-24

**Authors:** Muralidharan Sargurupremraj, Sathyaseelan Chakkarai, Yinan Zheng, Habil Zare, Xueqiu Jian, Qiong Yang, Jayandra Jung Himali, Sudha Seshadri

**Affiliations:** ^1^ University of Texas Health Science Center at San Antonio, San Antonio, TX, USA; ^2^ Northwestern University Feinberg School of Medicine, Chicago, IL, USA; ^3^ The University of Texas Health Science Center at San Antonio, San Antonio, TX, USA; ^4^ Boston University School of Public Health, Boston, MA, USA; ^5^ Department of Biostatistics, Boston University School of Public Health, Boston, MA, USA; ^6^ Glenn Biggs Institute for Alzheimer's & Neurodegenerative Diseases, University of Texas Health San Antonio, San Antonio, TX, USA; ^7^ Department of Neurology, Boston University School of Medicine, Boston, MA, USA; ^8^ Glenn Biggs Institute for Alzheimer's & Neurodegenerative Diseases, University of Texas Health Science Center, San Antonio, TX, USA; ^9^ Framingham Heart Study, Framingham, Boston, MA, USA; ^10^ Department of Neurology, University of Texas Health Sciences Center, San Antonio, TX, USA, San Antonio, TX, USA

## Abstract

**Background:**

Transposable elements (TEs) are repetitive DNA sequences that make up about 45% of the genome and capable of autonomous relocation. While in‐vivo animal models have mechanistically linked TEs to Alzheimer's disease (AD) pathology, the vast diversity of TE families in humans makes it challenging to study their role on a population‐wide scale. In this study, we jointly considered genomic and epigenetic (methylation) data, to provide insights on how TEs influence the preclinical processes of ADRD that are measurable as brain‐imaging endophenotypes and AD risk.

**Method:**

Leveraging multi‐omics data from the Trans‐Omics for Precision Medicine Program (TOPMed) and Accelerating Medicines Partnership Program for AD (AMP‐AD) initiatives, along with machine‐learning algorithms; we genotyped (i) polymorphic TE insertions (pTEIs), and (ii) predicted methylation state of TEs across different families (LINE, SINE, endogenous retrovirus or ERVs). Using whole‐genome regression model, we tested the genetic susceptibility conferred by pTEIs on hippocampal volume (HV) and total brain volume (TBV) (*N* = 2,472) within the Framingham Heart Study subset of TOPMed. Additionally, through functional enrichment models we studied differential methylation pattern of TEs in post‐mortem AD brains compared to cognitively normal controls within AMP‐AD cohorts.

**Result:**

Common pTEI (minor allele frequency >5%) from the highly active LINE‐1 (L1Hs) family, located in the promoter region of *RYR3* ‐ a gene implicated in amyloid‐β production ‐ was significantly associated with TBV atrophy in cognitively normal individuals. Functional enrichment analysis further revealed altered methylation patterns of TEs from the same L1Hs family in AD brains compared to controls. Notably, TEs within *KDM2B*, a key epigenetic regulator of chromatin state, showed differential methylation; lower *KDM2B* levels are known to disrupt hippocampal morphogenesis and accelerate cognitive decline. Interestingly, our region‐based burden test aggregating the phenotypic effects of multiple pTEIs identified *SPATA5*, a gene involved in chromatin accessibility, as significantly associated with HV.

**Conclusion:**

By integrating genomic and methylation data, we offer novel insights into the role of TEs in AD pathogenesis and their contribution to increased disease risk. Our ongoing work, leveraging ChIP‐seq data on DNA accessibility (chromatin state), aims to further characterize these latent regulatory elements and their impact on gene regulation in a context‐specific manner.

